# Management of malignant pleural effusion and ascites by a triple access multi perforated large diameter catheter port system

**DOI:** 10.1186/1477-7819-6-85

**Published:** 2008-08-18

**Authors:** Ihsan Inan, Sandra De Sousa, Patrick O Myers, Brigitte Bouclier, Pierre-Yves Dietrich, Monica E Hagen, Philippe Morel

**Affiliations:** 1 Visceral Surgery Division, Department of Surgery, Geneva University Hospital, Rue Micheli-du-Crest 24, CH-1211, Geneva, Switzerland; 2 Oncology Department, Geneva University Hospital, Rue Micheli-du-Crest 24, CH-1211, Geneva, Switzerland

## Abstract

**Background:**

Pleural or peritoneal effusions (ascites) are frequent in terminal stage malignancies. Medical management may be hazardous.

**Methods:**

A 60-year-old man with metastatic malignant melanoma presented refractory ascites as well as bilateral pleural effusions. After failure of the medical treatment, bilateral pleural aspiration and paracentesis became necessary two to three times a week. A multi perforated 15F silicone catheter connected with a subcutaneous port was implanted in peritoneal and both pleural cavities surgically under general anesthesia. Leakage around the catheter is prevented by subcutaneous tunneling. Surgical technique is described and illustrated in a video.

**Results:**

Implanted systems were immediately operational. Follow up period was 41 days. Each port was accessed 10 times and a total of 65'200 ml of fluid was drained. By the end of the forth week, pleural effusions diminished, systems were controlled for permeability and chest x-rays confirmed absence of effusion.

**Conclusion:**

Implanted port systems for refractory ascites and pleural effusions avoid morbidity and the patient's anxiety related to repeated puncture-aspiration. Large catheter diameter allows an easy and fast drainage of large volumes. Compared to chronic indwelling catheters, subcutaneous location of port system allows an entire integration, giving the patient a total liberty in daily life between two sessions of drainage. Drainage can be performed in an outpatient basis as an ambulatory procedure. This patient-friendly technique may be a treatment option in case of failure of other techniques.

## Background

Pleural effusion and ascites are frequent in terminal stage malignancies. In the United States, patients affected by malignant pleural effusions alone is estimated to 175'000 per year [1]. Fluid sequestration significantly compromises patient's quality of life.

Almost 75% of all malignant pleural effusions are due to malignancies of breasts, lungs, ovaries and lymphomas. Malignant pleural effusions account for approximately 40% of chronic pleuritis cases. They are mostly recurrent and often resistant to systemic treatment. They occur mainly from obstruction or disruption of lymphatic channels by malignant cells.

In case of symptomatic malignant pleural effusion, dyspnea (moderate to severe, according to the importance of the effusion), cough, thoracic discomfort as well as pain may be present [2].

Malignant ascites leads to shortness of breath, nausea, diminished appetite and early satiety, fatigue, lower extremity edema, limited mobility and difficulty to fit clothes. Ascites results from multiple mechanisms including vascular permeability changes, peritoneal carcinomatosis (metastatic implants of carcinoma on the peritoneal cavity), lymph drainage obstruction, hepatic congestion due to tumour infiltration or neoplastic production of exudative fluid [3]. Ascites may develop in various circumstances but mainly in cirrhosis and peritoneal carcinomatosis. Complication may arise, such as respiratory restriction and respiratory distress under diaphragmatic compression (elevation of diaphragm, compressing the lung and reducing their compliance), spontaneous bacterial peritonitis, electrolyte and hemodynamic disturbances, hepatorenal syndrome, physical discomfort with limitation of the movements leading to reduction of the quality of life [4,5].

The aim of treatment is to improve the quality of life by decreasing these symptoms. We report our experience with a patient presenting both pleural and peritoneal effusions. Multiperforated large diameter, totally implanted port systems were surgically inserted in each cavity. The clinical course of the patient is summarized, treatment options discussed and surgical technique is described in a video file.

## Clinical experience

A 60-year-old man known for a malignant melanoma since 1999 developed a small bowel metastasis in 2003. Since he was detected as HIV, stage III A+ in 2002, he was not integrated in specific immunotherapy programs and no other treatment was proposed. In 2005, he presented with refractory ascites as well as bilateral pleural effusions. Patient refused any kind of pleurodesis. A central venous access port was implanted and he received three cycles of chemotherapy (Vinblastine, Dacarbazine and Cisplatine). This measure also failed and the patient required peritoneal paracentesis and thoracentesis 2 to 3 times a week. In 2006, the patient was referred by his oncologist and the decision was made to insert multiperforated large diameter, totally implanted port systems into the peritoneal and both pleural cavities. Chronic indwelling pleural or peritoneal catheter systems available (PleurX™) were discussed and not retained neither by the patient nor by oncologist because of inability of the patient to learn to manage systems at the end stage of malignancy. Access system implantationPatient informed about the technique before scheduling the operation. Procedure was realized under standard balanced general anaesthesia (Additional files 1, 2, and 3).

**Figure 1:**
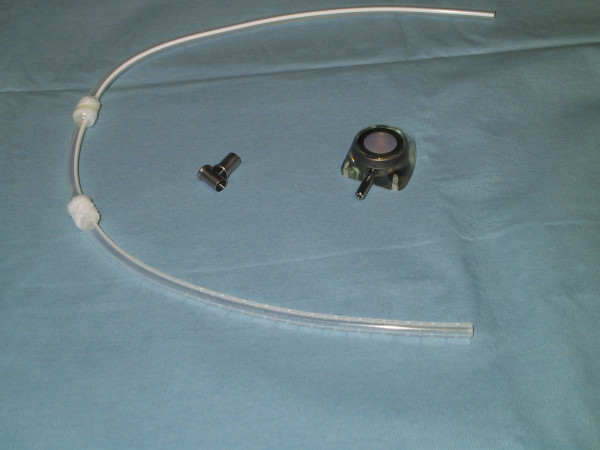
**Multi perforated large diameter silicone catheter port system with two Dacron cuffs**.

**Figure 2:**
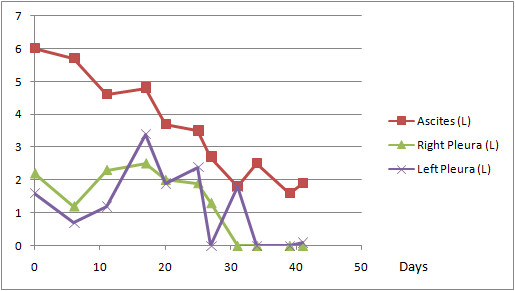
**Evolution of ascites and pleural fluid volume drained**.

A Celsite T203J (B. Braun Medical SA, Sempach, Switzerland) port system with multi perforated large diameter silicone catheter (outer diameter: 4.9 mm, inner diameter: 2.6 mm) with two Dacron cuffs (Figure 1) was first implanted into the peritoneal cavity by a muscle splitting transrectal incision. The catheter was positioned in the right paracolic space. Patient received 20 g. of albumin per two litres of peritoneal and pleural effusion. Volume replacement and hemodynamic status monitored by anesthesia team stayed stable during whole procedure. A purse string suture was placed on the peritoneum and tied around the Dacron cuff to insure watertight sealing, in order to prevent leakage. A second incision was made on the right costal margin at the anterior axillary line and a 3-cm subcutaneous pocket created on the thoracic wall. The catheter was passed to the proximal incision with a tunnelling device and connected to the port. The reservoir was anchored on the anterior thoracic fascia by monofilament non-absorbable 2.0 sutures. Identical systems were placed in each pleural cavity with the same open surgical technique. A chest x-ray at the end of the procedure in the recovery room showed correct position of the catheters and no residual pneumothorax. The follow up period was 41 days. Each port was accessed 10 times and 65'200 ml of fluid was drained. Effusion drainage was carried out by using a peritoneal dialysis recipient system placed distally and connected to a 1.1 × 19 mm Huber needle. It lasted 2h30 on average for volumes ranging from 600 ml to 5'700 ml (Figure 2). By the end of the forth week, pleural effusions diminished, systems were controlled for permeability and chest x-rays confirmed absence of effusions. All care was given as a day procedure, without hospital stay; the patient continued his daily activities normally and maintained a good quality of life until his last days. Death occurred due to brain metastasis, 7 weeks after the implantation of the triple access system.

## Discussion

There are several attitudes to manage pleural and abdominal intracavitary refractory effusions in end stage patients [6]. The aim of the treatment is to improve quality of life by decreasing symptoms. First line treatment is therapeutic pleural aspiration. In case of relapse or delayed management, repetitive pleural aspiration may be necessary. Repeated pleural aspiration may be complicated by pneumothorax, bleeding, infection and spleen or liver laceration [7]. Pleurodesis, by mini thoracotomy or thoracoscopy is favoured in patients with limited survival [8]. Talc powder is preferred to other pleurodesis agents like bleomycine, tetracycline or doxyciline, a tetracycline analogue. Although unusual, effusion recurrence is possible early after pleurodesis, aspecially in high volume pleural effusions. When initial pleurodesis fails, there are several alternatives to consider: repeated pleurodesis, repeated pleural aspiration, systemic chemotherapy when tumours are likely to respond to such a treatment, pleuroperitoneal shunting or pleurectomy. Surgical procedures include parietal pleurectomy, or decortications. Unfortunately, for different reasons, some patients may not profit timely or do not benefit of pleurodesis. These patients suffer both from compressive effect of effusions between drainage sessions and from the risks and various complications of repeated pleural punctures. In case of failure or impossibility of pleurodesis, chronic indwelling intercostal catheter implantation is described as an alternative. An implantable port system with multi perforated large diameter catheter in the pleural cavity may be a treatment alternative for end stage patients [9,10].

Ninety percent of patients with ascites respond to standard medical therapies, such as diuretics, sodium and water restriction and diet. When ascites becomes chronic and refractive to medical treatment, various possibilities are available, such as aggressive diuretic therapy, high-volume paracentesis, ascites recirculation with peritoneovenous or intrahepatic portosystemic shunts [11]. Morbidity related to repeated abdominal puncture and paracentesis is well described, such as unsuccessful puncture, pain, infection or even septicaemia and haemorrhage [12]. Frequent large volume paracentesis require multiple visits to the healthcare facilities during the few remaining months of life [13]. A peritoneovenous LeVeen shunt may be complicated by pulmonary edema, presents poor permeability at long term and may be complicated by peritoneal fibrosis. Use of multi perforated large diameter catheter with implantable port systems for refractory ascites has several advantages. Large diameter of the catheter allows an easy and fast drainage of large volumes. Compared to chronic indwelling catheter systems, subcutaneous location of port system allows an entire corporeal integration, giving the patient a total liberty in daily life between two drainage sessions. Dacron cuffs placed on the catheter insures hermetic sealing of the host cavity and forms a barrier against infections.

## Conclusion

In conclusion, in this particular case, managing malignant pleural and peritoneal effusions with implanted large diameter multiperforated port systems was successful. This patient-friendly technique may be a treatment option in case of failure of other treatment options.

## Competing interests

The authors declare that they have no competing interests.

## Authors' contributions

II and SDS carried out the surgical care of the patient and the follow-up during the treatment, realised the illustration and drafted the manuscript. POM, SDS, PYD, MH participated to manuscript draft and literature research. BB, PYD participated in the follow-up of the patient as well as manuscript draft on oncologic aspect. MEH participated to manuscript draft and literature research. PM encouraged the case report, participated in its preparation and helped to draft the manuscript. All authors read and approved the final manuscript.

## Supplementary Material

Additional file 1 Click here for file

Additional file 2 Click here for file

Additional file 3 Click here for file
